# Characterization of a mGluR5 Knockout Rat Model with Hallmarks of Fragile X Syndrome

**DOI:** 10.3390/life12091308

**Published:** 2022-08-25

**Authors:** Victoria Dahl, Hawley Helmbrecht, Ana Rios Sigler, Kate Hildahl, Holly Sullivan, Sanjana Janakiraman, Saahiti Jasti, Elizabeth Nance

**Affiliations:** 1 Department of Chemical Engineering, University of Washington, Seattle, WA 98195, USA; 2 Department of Bioengineering, University of Washington, Seattle, WA 98105, USA; 3 Paul Allen School of Computer Science and Engineering, University of Washington, Seattle, WA 98195, USA; 4 Department of Biology, University of Washington, Seattle, WA 98195, USA; 5 Center on Human Development and Disability, University of Washington, Seattle, WA 98195, USA

**Keywords:** neuroinflammation, microglia, autism spectrum disorders (ASDs), neurodevelopmental disorders, extracellular matrix, machine learning

## Abstract

The number of reported cases of neurodevelopmental disorders has increased significantly in the last few decades, but the etiology of these diseases remains poorly understood. There is evidence of a fundamental link between genetic abnormalities and symptoms of autism spectrum disorders (ASDs), and the most common monogenetic inheritable form of ASDs is Fragile X Syndrome (FXS). Previous studies indicate that FXS is linked to glutamate signaling regulation by the G-protein-coupled metabotropic glutamate receptor 5 (mGluR5), which has been shown to have a regulatory role in neuroinflammation. We characterized the effect of knocking out mGluR5 in an organism known to have complex cognitive functions—the rat. The heterozygous phenotype is the most clinically relevant; therefore, we performed analysis in heterozygous pups. We showed developmental abnormalities in heterozygous mGluR5 knockout rats, as well as a significant increase in chemokine (C-X-C motif) ligand 1 (CXCL) expression, a hallmark indicator of early onset inflammation. We quantified an increase in microglial density in the knockout pups and quantified morphological phenotypes representative of greater reactivity in the male vs. female and postnatal day 28 heterozygous pups compared to postnatal day 14 heterozygous pups. In response to injury, reactive microglia release matrix metalloproteases, contribute to extracellular matrix (ECM) breakdown, and are responsible for eradicating cellular and molecular debris. In our study, the changes in microglial density and reactivity correlated with abnormalities in the mRNA expression levels of ECM proteins and with the density of perineuronal nets. We saw atypical neuropsychiatric behavior in open field and elevated plus tests in heterozygous pups compared to wild-type litter and age-matched controls. These results demonstrate the pathological potential of the mGluR5 knockout in rats and further support the presence of neuroinflammatory roots in ASDs.

## 1. Introduction

The number of diagnosed cases of autism spectrum disorders (ASDs) has grown dramatically in the past few decades, with identified ASD cases more than doubling from a 6.7% prevalence estimate in 2000 to 23% in 2018 [1,2]. Children diagnosed with ASDs experience intellectual disabilities, extreme social anxiety, tactile avoidance, and other symptoms that can impair development [3]. Additionally, males are more commonly diagnosed with ASDs than females [4]. Potential factors or causes of ASDs include genetic components such as a high frequency of reports of single-nucleotide polymorphisms and other genetic abnormalities in patients exhibiting symptoms of ASDs [5]. This indicates the potential of genetic therapies for ASDs [6], although the specific genes directly responsible for ASDs remain unknown [7]. 

Fragile X Syndrome (FXS) is the most common monogenetically inherited form of autism, which is characterized by a mutation that disrupts synaptic protein synthesis [8]. The mutation results in the inactivation of the fragile X mental retardation protein (FMRP) and developmental challenges common to ASDs [9]. Previous studies indicate that FMRP is regulated by the G-protein-coupled metabotropic glutamate receptor 5 (mGluR5), and inactivation of FMRP leads to impairment of complex brain functions [10]. mGluR5 has also been shown to regulate glutamate release and uptake in astrocytes [11] and has been implicated as a regulator of N-methyl-D-aspartate (NMDA) receptor activities, a specific type of ionotropic glutamate receptor [12]. Intracellular mGluR5 activates many symptoms associated with FXS, including weakened neuronal connections and dysregulated protein synthesis [10], indicating that blocking of mGluR5 is a potential therapeutic for FXS. Total gene knockout mouse models indicate mGluR5 can downregulate microglial activation and neuroinflammation [13]. Thus, research on the functional effects of mGluR5 and how it is distributed within the cell can aid in the development of preventative treatment for FXS.

While all previous studies have used mice or zebrafish as animal models of FXS or ASDs, we explored an mGluR5 knockout model in rats, which have more complex social and cognitive function than mice [14]. Given that mGluR5 signaling has a regulatory role in neuroinflammation [15], we used the mGluR5 knockout rat to analyze homozygous and heterozygous knockout-associated alteration in microglial density and morphology, compared to litter and age-matched wild-type pups. We further assessed the associated effects on inflammatory cytokines, extracellular matrix (ECM) expression profiles, the density of perineuronal nets (PNNs), and neuropsychiatric abnormalities. We place emphasis on characterizing the heterozygous knockout to increase clinical relevance, as the actual incidence of total gene knockout in humans is low [16].

## 2. Materials and Methods

### 2.1. Animals and Genotyping

The selection and treatment of experimental animals and animal handling procedures were followed in accordance with protocols approved by the Institutional Animal Care and Use Committee (IACUC) (Protocol 4383-01) at the University of Washington and NIH guidelines. The animals used in the study were an established colony of mGluR5 knockout rats originating from Sigma Advanced Genetic Engineering (SAGE) labs (Sigma, St. Louis, MO, USA), with a breeding license purchased by UW from SAGE. These genetically modified transgenic Sprague Dawley pups have a biallelic deletion of the metabotropic glutamate receptor 5 (mGluR5). The animals were bred in-house, beginning at 8–12 weeks of age. HET females were bred a total of 2 times and then retired. Pups were weaned at postnatal day 21 (P21) and placed in sex-segregated cages. Animals were identified using a 1 mm ear hole punch performed at P10-P21 prior to weaning. Ear punch location correlated to a standard ID system for the pups. Animals were kept on a 12 h light/12 h dark cycle and were euthanized at specified study end points during the first six hours of the light phase. 

For genotyping, tissue lysates were obtained from tail clips taken from P7 rat pups. Tail pieces were immersed in 1200 μL SNET warmed to 55 °C and 30 μL Proteinase K and allowed to digest at 55 °C for a minimum of 3 h. Samples were vortexed approximately every 20 min during the digestion period. Phenol chloroform was added to the samples and then centrifuged at 25 °C and 15,000× *g* for 3 min. The supernatant was removed and mixed with chilled isopropyl alcohol and centrifuged at 4 °C and 15,000× *g* for 25 min. The supernatant was removed, and the remaining pellet of DNA was washed with chilled 70% ethanol. The remaining solution was allowed to evaporate, and the pellet was resuspended in 50 μL real-time polymerase-chain-reaction (RT-PCR) -grade water per sample and vortexed to reconstitute DNA. The concentration of DNA was quantified to ensure adequate yield and purity, and RT-PCR was run using iTaq Universal Sybr Green Forward and Reverse primers (Thermo Fisher, Waltham, MA, USA). Genotypes were identified based on melting temperature and melting peaks.

### 2.2. Tissue Preparation

At the appropriate time point, animals were euthanized using a fatal pentobarbital overdose (0.1 mL of pentobarbital per 50 g body weight). At P28, euthanasia was performed after behavioral testing was complete. After injection, a transcardial perfusion was performed with Dulbecco’s 1x phosphate-buffered saline (PBS, Corning, Corning, NY, USA), and the brain was removed. One hemisphere was fixed in 10% phosphate-buffered formalin solution (Fisher Scientific, Hampton, NH, USA) to be used for immunofluorescence analysis and the other stored in RNALater for RNA expression. 

### 2.3. Antibodies

The primary antibody rabbit anti-Iba (019-19741, Wako Fujfilm, Richmond, VA, USA) was prepared in a solution of PBS (Corning, Corning, NY, USA), 1% Triton X-100 (Sigma, St Louis, MO, USA), and goat serum (Thermo Fisher, Waltham, MA, USA), referred to as PBS-g++. The secondary antibody goat anti-rabbit IgG conjugated to AlexaFluor488 (Fisher Scientific, Hampton, NH, USA) was prepared in PBS+, a solution containing PBS and 1% Triton X-100 (Sigma, St Louis, MO, USA).

### 2.4. Immunofluorescence Staining

Formalin-fixed brains were cryoprotected with a 30% sucrose solution for a minimum of 24 h. The brains were frozen at −20 °C and cryosectioned coronally into 30 μm slices and mounted on gelatin-coated slides. The consistency of depth was maintained by collecting slices at the first appearance of the dorsal hippocampus in the sagittal plane while making slices beginning at the anterior of the brain. A total of fifty-four 30 μm slices were obtained from each brain.

Primary antibody was prepared in PBS-g++ at a 1:250 ratio. Five-hundred microliters of this solution was added to formalin-fixed tissue slices and left in a dark and humid environment for 6 h. The slides were washed 2 times with 1x PBS, then 250 μL of the secondary antibody solution, prepared at a 1:500 ratio (antibody:PBS+), was added and incubated for 2 h in a dark and humid environment. Each slide was again washed 2x in 1x PBS, and a DAPI solution (999 μL 1x PBS and 1 μL 5 mg/μL DAPI stock) was added to each slide for 15 min to stain cell nuclei.

### 2.5. Microglia Quantification

Slides and images were coded, and the analysis was performed with the personnel blinded to the experimental groups. Confocal images (x63; 4–6 images/animal) were acquired from the cortex, hippocampus, and thalamus using Nikon A1R (Nikon, Melville, NY, USA). The confocal images were exported into individual channels using NIS-Elements software (Nikon, Melville, NY, USA). The Iba-1+ (green) cells were counted and averaged to the area of the region of interest. Analyses were performed in a manner blinded to the treatment group to minimize any bias.

### 2.6. PNN Quantification

Formalin-fixed brains were sliced and mounted in the same method as described for microglia quantification. A Wisteria floribunda agglutinin (WFA) lectin stain was applied for a 2 h incubation time, and slides were washed with a PBS solution. Each slide was again washed 2x in 1x PBS solution, and a DAPI solution (999vμL 1x PBS and 1 μL 5 mg/μL DAPI stock) was added to each slide for 15 min to stain cell nuclei. Slides and images were coded, and the analysis was performed with the personnel blinded to the experimental group.

### 2.7. RT-PCR Cytokine Expression

Brains were extracted and placed in RNAlater solution for storage before RNA extraction. The brain tissue was weighed and then homogenized in 1 mL TRIzol per 100 mg tissue. Separation of DNA and proteins was then performed using 200 μL chloroform per 100 mg tissue and centrifugation. Once the RNA was isolated, a series of wash steps using isopropyl alcohol and ethanol was performed to precipitate pure RNA. The purity of the RNA samples was assessed using Nano Drop (ND-1000, Thermo Fisher, Waltham, MA, USA). Reverse transcription was performed using 2500 ng total RNA and 4 μL of reagent (SuperScript™ IV VILO™ (SSIV VILO) Master Mix, Thermo Fisher, Waltham, MA, USA). Primers were annealed for 10 min at 25 °C, incubated for 10 min at 50 °C, and then at 95 °C for 5 min. The volume of cDNA was diluted to obtain 100 μL of 20 ng/μL cDNA. Once the cDNA was isolated, qRT-PCR was performed with Fast SYBR Green Master Mix (Thermo Fisher, Waltham, MA, USA) by a StepOnePlus RealTime PCR System. iTaq Supermix, cDNA, and forward/reverse primers for each respective cytokine were prepared and kept on ice with a total reaction mix volume of 10μL including 20 ng of cDNA. The reactions were loaded into triplicate wells and loaded into a CFX RT-PCR system. The expression of GAPDH mRNA was used to normalize target gene expression levels, and the P14 age group of mGluR5+/+ was used as the control for comparative cycle threshold (2^−ΔΔCt^) calculations. All gene expression levels were normalized and are expressed as the relative fold change to the housekeeping gene of the control condition.

### 2.8. Elevated Plus and Open Field Behavioral Testing

Behavioral testing was performed on pups when they reached P28. Behavioral testing for all animals was performed during the first six hours of the light phase of a 12 h light/12 h dark cycle. Animals were moved into a dedicated behavioral testing room, and cages were covered with a dark cloth for 5 min prior to testing to minimize the anxiety introduced by a novel environment. An elevated plus maze with two open and two closed arms was located beneath a motion-sensitive camera. The maze was constructed with dark opaque polyethylene; open arms had dimensions of 25 × 5 × 0.5 cm, the closed arms 25 × 5 × 16 cm, and the center platform 5 × 5 × 0.5 cm. At the beginning of each trial, pups were placed in the center of the elevated plus maze facing the same direction each time. The elevated plus maze was sanitized between each trial. EthoVision XT 14 software (Noldus, Leesburg, VA, USA) was used to track the movement of the nose-point, the movement of the center-point, the velocity, and the location of both the nose- and center-point of the animals over a 5 min time period. The analytical version of the software was used to calculate average time spent in open vs. closed arms, average center-point velocity, and total distance moved by the animals [17]. Open field behavioral testing was performed in a similar fashion. An open field-testing maze was placed underneath a motion-sensitive camera and was used to track the activity of the animals over a 5 min trial. A square open field testing maze was used with dimensions of 1.2 m × 1.2 m with a wall height of 50 cm. Animals were placed in the middle of the open field maze facing the same direction, and the EthnoVision software was again used to track and analyze their movement [18].

### 2.9. Cell Morphology Analysis

All images of interest from each group, sex, and age of Iba-1-stained slices were converted from the Nikon Elements’s .nd2 file format to .tiff file format using ImageJ’s batch converter. The saved .tiff files of all images were segmented by multiple thresholding methods using scikit-image’s try_all_thresholds functionality in Python, which includes the Isodata, Li, mean, minimum, Otsu, triangle, and Yen thresholds [19]. An image showing the original cell image with each threshold method was saved for qualitative comparison. The mean threshold method for segmentation was determined to be the most accurate based on qualitative cell count, branch segmentation, and best accuracy in differentiating overlapping cells. For final threshold-based segmentation, a gaussian blur with a sigma of 0.25 was applied to all images followed by the implementation of the scikit-image threshold_mean function. After segmentation, all small objects were removed, holes were filled, and all cells touching the border of the image were removed. We determined a pixel size of 265 for removing small objects with the following process: we determined from the literature that the approximate size of microglia is 1600 μm^2^ [20]; to ensure we did not exclude any microglia, we selected a lower boundary for size exclusion of 800 μm^2^, then we converted to pixels using the confocal microscope provided conversion of 0.575 pixel width = 1 μm conversion for the 60x magnification images. Cells touching the border of the image were removed since they are not full representations of cells and add noise to the data due to artificial shapes caused by the edge of the images.

After segmentation, cells were quantified with two methods: regionprops analysis with scikit-image and with Visually Aided Morpho-Phenotyping Image Recognition (VAMPIRE) [21]. With the regionprops functionality, cells were individually measured for geometric parameters including, area, convex area, perimeter, and solidity, where solidity is the ratio of pixels in the region to the region of pixels in the convex hull. Circularity was calculated according to the following equation:

$$Circularity=\frac{4\pi *Area}{Perimeter^{2}}$$

A circularity value of 1 is a perfect circle, and a value approaching 0 grows increasingly ellipsoid.

For VAMPIRE analysis, the Numpy files of the cell images after segmentation with the mean threshold were split by group of interest into an 80:20 train:test split. The training group was used to build a model with 50 coordinate points and 5 shape modes (SMs). The number of SMs was chosen to capture biological variability in the microglia while remaining computationally efficient. The model was then applied separately to each test group containing the remaining 20% of the data. The VAMPIRE method also produces results for each SM-sorted cell and their related area, perimeter, circularity, and aspect ratio. Qualitative visualizations of the segmented cells and their assigned shape modes were created with Python by assigning each shape mode a color with the Matplotlib twilight color map [22]. Heat maps of shape mode frequencies were created in Microsoft Excel with conditional formatting and green-white-purple color scales. The variance was calculated using the Excel “VAR” function for each group. All code can be found at: https://github.com/Nance-Lab/cellmorphflows/tree/master/Dahl_Mglur5, last accessed 24 June 2022 [23].

### 2.10. Statistical Analysis

Data were statistically analyzed by unpaired Student’s *t*-test to compare the means between unpaired samples, and Welch’s correction was applied in the case of unequal variance. *p* < 0.05 was considered to be statistically significant, and * *p* < 0.05, ** *p* < 0.01, *** *p* < 0.001, and **** *p* < 0.0001. F-tests were used to determine significant differences between population variances. Statistical analysis was run using the GraphPad Prism Software (Prism 8, San Diego, CA, USA).

For the microglial morphology analysis, plots were created and statistical analysis performed in GraphPad Prism Version 9.4.0 (San Diego, CA, USA). Graphs are displayed as the median with the interquartile range, and all data points are shown. Features were compared for individual microglial cell analysis and for SMs vs. geometric feature across groups using a non-parametric one-way ANOVA with the Kruskal–Wallis test and Dunn’s post hoc correction for multiple comparisons. All *p*-values < 0.05 were considered statistically significant. For the individual features calculated with regionprops, the decision to use the Kruskal–Wallis test for non-parametric data was made by testing the normal distribution of the data. The normality and lognormality distribution of each group and feature was tested using four tests: the D’Agostino and Pearson test, the Anderson–Darling Test, the Shapiro–Wilk test, and the Kolmogorov–Smirnov test. All experimental groups failed each normality test with a *p*-value < 0.001. The normal QQ plots were created in GraphPad Prism to show the deviation from a normal distribution.

## 3. Results

### 3.1. Assessment of Developmental Abnormalities in the mGluR5 Knockout Model in Rats

**Figure 1:**
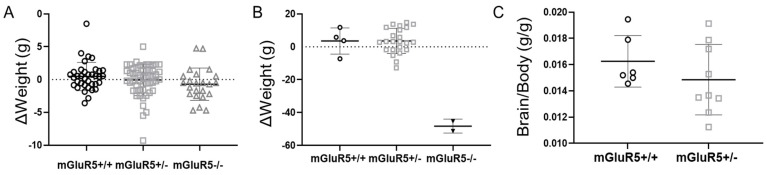
(**A**) At P14, deviation from average litter-matched body weight for both sexes and mGluR5^+/+^ (n = 37), mGluR5^+/−^ (n = 57), and mGluR5^−/−^ (n = 23) genotypes. (**B**) At P28, deviation from average litter-matched body weight for both sexes and mGluR5^+/+^ (n = 4), mGluR5^+/−^ (n = 23), and mGluR5^−/−^ (n = 2) genotypes. (**C**) Brain-to-body-weight ratio comparing mGluR5^+/−^ (n = 6) and mGluR5^+/+^ (n = 9) for sex-aggregated P14 pups. Error bars are the mean ± SD.

The normalized body weights of the wild-type (WT, mGluR5^+/+^), heterozygous (HET, mGluR5^+/−^), and knockout (KO, mGluR5^-/-^) phenotypes were compared for both P14 and P28 pups. Due to high variance in body weight between litters, the data are reported as the deviation from the average weight of each litter (Figure 1A,B). Of note, significant developmental abnormalities were observed in the mGluR5^−/−^ pups, and a very low number survived to the P28 age (Figure 1B); most available mGluR5^+/+^ pups were used for assessments at P14, and only surviving litter-matched pups were used for P28 assessment. Brain-to-body-weight ratios at P14 were calculated for each genotype (Figure 1C). Baseline levels of health were established by comparison of body weights in the experimental litter with control pups from non-genetically modified Sprague Dawley rats for the P14 age group (Figure S1) and compared based on sex (Figure S2). The average brain weights of P14 pups were 0.915 +/- 0.134 g, well within physiological standards for the Sprague Dawley rat at this age [24,25].

### 3.2. Inflammatory Cytokine Expression Profiles in mGluR5^+/+^ and mGluR5^+/-^ Genotypes

**Figure 2:**
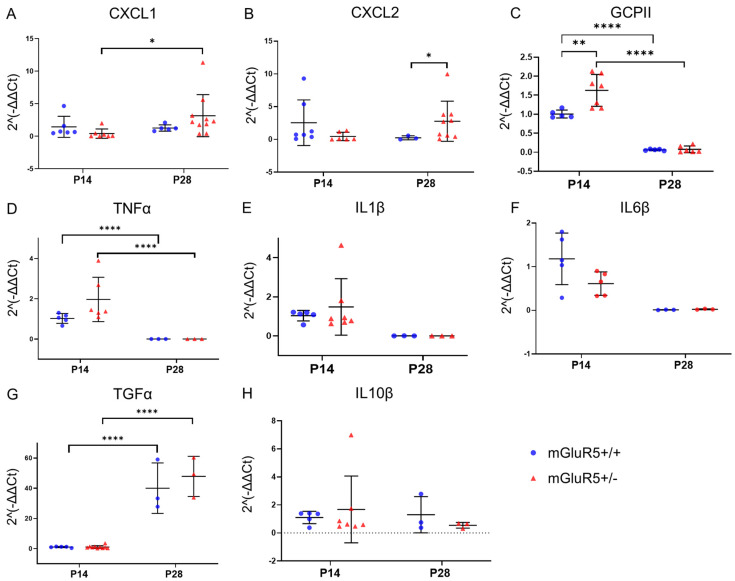
Gene expression for both P14 and P28 mGluR5^+/+^ and mGluR5^+/^^−^ pups are depicted for: (**A**) CXCL1 (n = 6 P14 mGluR5^+/+^, n = 7 P14 mGLuR5^+/^^−^, n = 5 P28 mGluR5^+/+^, n = 10 P28 mGluR5^+/^^−^); (**B**) CXCL2(n = 6 P14 mGluR5^+/+^, n = 7 P14 mGLuR5^+/^^−^, n = 5 P28 mGluR5^+/+^, n = 10 P28 mGluR5^+/^^−^); (**C**) GCPII (n = 5 P14 mGluR5^+/+^, n = 6 P14 mGLuR5^+/^^−^, n = 3 P28 mGluR5^+/+^, n = 3 P28 mGluR5^+/^^−^); (**D**) TNFα (n = 5 P14 mGluR5^+/+^, n = 7 P14 mGLuR5^+/^^−^, n = 5 P28 mGluR5^+/+^, n = 6 P28 mGluR5^+/^^−^); (**E**) IL1β (n = 5 P14 mGluR5^+/+^, n = 7 P14 mGLuR5^+/^^−^, n = 3 P28 mGluR5^+/+^, n = 3 P28 mGluR5^+/-^); (**F**) IL6β (n = 5 P14 mGluR5^+/+^, n = 5 P14 mGLuR5^+/^^−^, n = 3 P28 mGluR5^+/+^, n = 3 P28 mGluR5^+/^^−^); (**G**) TGFα (n = 5 P14 mGluR5^+/+^, n = 7 P14 mGLuR5^+/^^−^, n = 3 P28 mGluR5^+/+^, n = 3 P28 mGluR5^+/^^−^); and (**H**) IL10β (n = 5 P14 mGluR5^+/+^, n = 7 P14 mGLuR5^+/^^−^, n = 3 P28 mGluR5^+/+^, n = 6 P28 mGluR5^+/^^−^). All gene expression results are normalized to mGluR5^+/+^ pups. Blue represents mGluR5^+/+^ and red represents mGluR5^+/^^−^.* *p* < 0.05, ** *p* < 0.01, and **** *p* < 0.0001.

mRNA expression levels of chemokine (C-X-C motif) ligand 1 (CXCL1) and CXCL2 were compared for both mGluR5^+/+^ and mGluR5^+/-^ genotypes for the P14 and P28 age groups (Figure 2A,B). When comparing gene expression for CXCL1, there was a 7.75-fold decrease in the 2^−ΔΔCt^ values in P14 vs. P28 pups for the mGluR5^+/-^ genotype, with a *p*-value of 0.0434 (Figure 2A). This decrease was not observed across the mGluR5^+/+^ age groups. When comparing the coefficient of variation for the mGluR5^+/+^ vs. mGluR5^+/-^ P14 pups, it was found that the mGluR5^+/-^ population had a coefficient of variation (CV) of 25.78 compared to a CV of 10.30 for the mGluR5^+/+^ population. An f-test showed statistically significant variance in the populations with a *p*-value of 0.0173. We also compared the CV for P28 pups and found the mGluR5^+/+^ population had a CV of 33.92, while the mGluR5^+/-^ population had a CV of 114.1. An f-test resulted in statistically significant differences in the variance of the two populations (*p*-value = 0.0167).

When comparing gene expression for CXCL2, there was a 6.2-fold decrease in values for mGluR5^+/+^ pups vs. mGluR5^+/-^ pups when comparing the P28 age group, with a *p*-value of 0.0391 (Figure 2B). A comparison of the f-distribution of variation for the mGluR5^+/+^ vs. mGluR5^+/-^ P14 pups showed statistically significant variance in the populations with a *p*-value of 0.0018. An f-test for the mGluR5^+/+^ vs. mGluR5^+/-^ P28 pups resulted in a statistically significant *p*-value of 0.0195, and a comparison of the P14 vs. P28 ages for the mGluR5^+/+^ and mGluR5^+/-^ groups had statistically significant *p*-values of 0.0152 and 0.0030, respectively.

We also tested mRNA expression of glutamate carboxypeptidase II (GCPII) for both genotypes and age groups. There was a 1.67-fold increase in the 2^−ΔΔCt^ values in mGluR5^+/-^ pups over mGluR5^+/+^ pups in the P14 age group with a *p*-value of 0.0094, but this was not sustained in the P28 age group (Figure 2C). There were also significant decreases in the values for P14 vs. P28 pups when comparing each respective genotype group, with *p*-values < 0.0001. For P14, the mGluR5^+/-^ population had a coefficient of variation (CV) of 25.78 compared to a CV of 10.30 for the mGluR5^+/+^ population. An f-test showed statistically significant variance in the populations with a *p*-value of 0.0173. We also compared the CV for P28 pups, and the mGluR5^+/+^ population had a CV of 33.92, while the mGluR5^+/-^ population had a CV of 114.1. An f-test resulted in a statistically significant difference in the variance of the two populations, with a *p*-value of 0.0167.

We further observed a 315-fold and 691-fold decrease in TNFα expression in P28 compared to P14 animals in mGluR5^+/+^ and mGluR5^+/-^ animals, respectively (Figure 2D). An f-test comparing the variance of the profiles in mGluR5^+/+^ and mGluR5^+/-^ animals in the P14 age group revealed significantly higher variance in the mGluR5^+/-^ profiles, with a *p*-value of 0.0121. When comparing gene expression of IL1β, IL6β, and IL10β, between mGluR5^+/-^ and mGluR5^+/+^, no statistically significant differences were seen between the groups for either the P14 or P28 age groups (Figure 2E,F,H). Gene expression of TGFα showed a statistically significant 40-fold (*p* = 0.0001) and 41-fold (*p* = 0.0001) increase in expression in the P28 age group for the mGluR5^+/-^ and mGluR5^+/+^ groups, respectively (Figure 2G).

### 3.3. Microglial Density in mGluR5^+/-^ Pups

**Figure 3:**
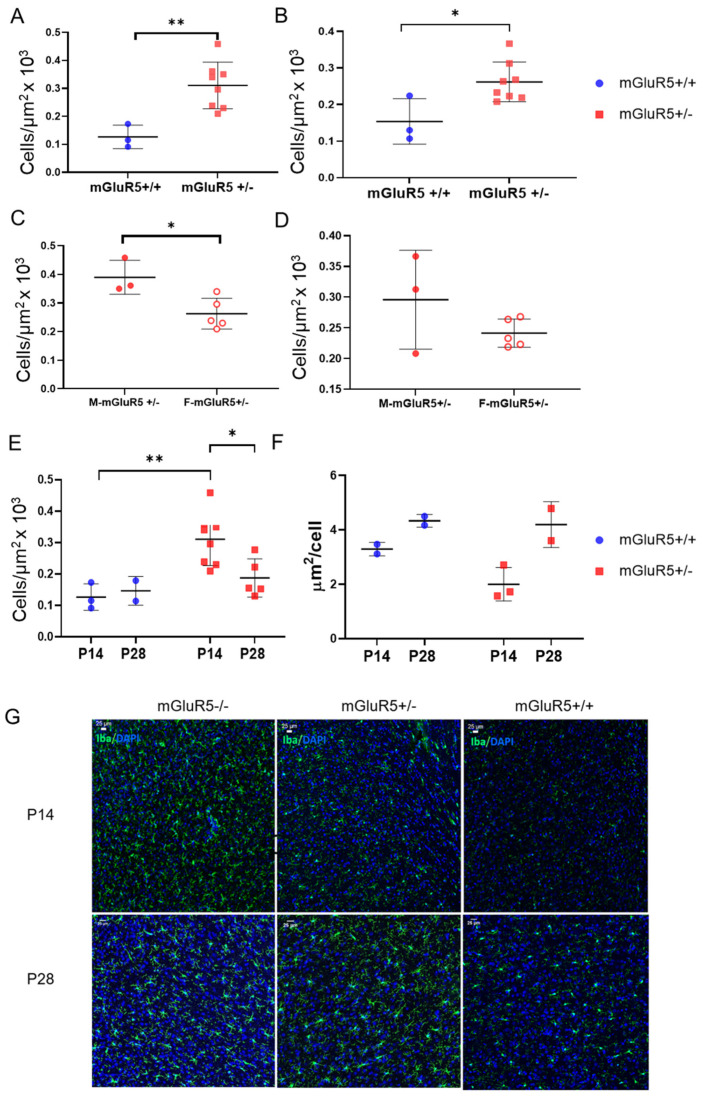
(**A**) P14 cortical microglia density in mGluR5^+/+^ (n = 3) and mGluR5^+/−^ (n = 8) pups (*p* = 0.0062). (**B**) P28 hippocampal microglia density aggregate mGluR5^+/+^ (n = 3) and mGluR5^+/−^ (n = 8) pups (*p* = 0.0193). (**C**) Comparison of microglia density in the cortex for male (n = 3) and female (n = 5) P14 mGluR5^+/-^ pups (*p* = 0.0206). (**D**) Comparison of microglia density in the hippocampus for male (n = 3) and female (n = 5) P14 mGluR5^+/−^ pups. (**E**) Microglia density in the cortex for P14 (n = 3) and P28 (n = 2) mGluR5^+/+^ and P14 (n = 8) and P28 (n = 5) mGluR5^+/−^ pups (*p* = 0.0161). (**F**) Area covered per cell detected for microglia in the cortex for P14 (n = 2) and P28 (n = 2) mGluR5^+/+^ and P14 (n = 3) and P28 (n = 2) mGluR5^+/−^ pups. (**G**) Representative images of microglia density in mGluR5^+/+^, mGluR5^+/^^−^, and mGluR5^−^^/−^ at P14 and P28. * *p* < 0.05, ** *p* < 0.01.

Microglia density was characterized in three brain regions, the cortex, the thalamus, and the hippocampus, to determine the associated impact of heterozygous mGluR5 knockout. The most significant trends were observed in the cortical microglia. A 2.5-fold increase was observed in microglia density in the cortex of mGluR5^+/-^ pups when compared to male mGluR5^+/+^ pups in the P14 age groups (*p* = 0.0223) (Figure 3A). This trend was also significant in the hippocampus when comparing male and female pups together (*p* = 0.0193) (Figure 3B). Additionally, average microglia density in the cortex was 382 cells per square micrometer in the male P14 mGluR5^+/-^ brain and 281 cells per square micrometer in the female P14 mGluR5^+/-^ brain (Figure 3C). This represented a significant increase (*p* = 0.0206) in microglia density in male mGluR5^+/-^ pups compared to female mGluR5^+/-^ pups.

A comparison of microglia density in the hippocampus for male and female P14 mGluR5^+/−^ pups was also performed and showed no significant difference between the groups (Figure 3D). A 1.7-fold decrease of average microglia density in the mGluR5^+/−^ pups was observed at P14 compared to P28 (*p* = 0.0161) (Figure 3E). This motivated an investigation regarding how the cells in these two age groups are different morphologically. To evaluate the morphological difference between cells in these age groups, the mean area covered per cell was determined to detect amoeboid vs. ramified properties. The average area covered per cell was found to increase from P14 to P28 for both mGluR5^+/+^ and mGluR5^+/^^−^ groups (Figure 3F). High-magnification images and qualitative evaluation of individual microglia morphology were also employed to help determine differences. Representative images of microglial cell density are presented in Figure 3G.

**Figure 4:**
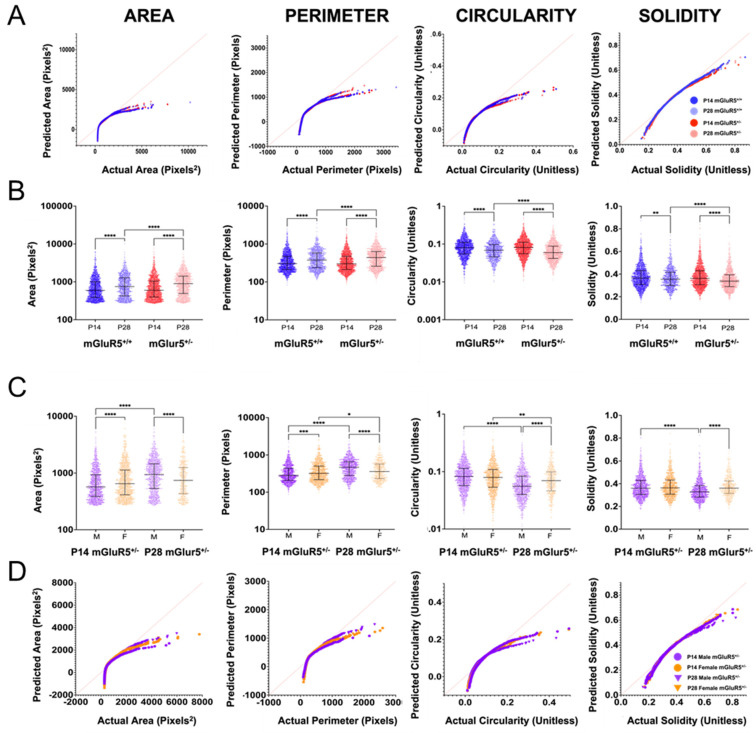
(**A**) Normal QQ plots of the area, perimeter, circularity, and solidity for the P14 and P28 mGluR5^+/+^ and mGluR5^+/−^ animals showing the distribution deviation from normality, represented by the red line through the origin. (**B**) Microglial geometric parameters for the mGluR5^+/+^ (blue) and mGluR5^+/−^ (red) groups at both P14 (darker intensity) and P28 (lighter intensity): area, perimeter, circularity, and solidity. There were 9 brain slices for P14 mGluR5^+/+^ and P28 mGluR5^+/−^; 6 brain slices for P28 mGluR5^+/+^; 12 brain slices for P14 mGluR5^+/−^. (**C**) Microglial geometric parameters for the P14 and P28 animals in both the males and females. There were 6 brain slices for each group except the P28 female mGluR5^+/−^, which used 3 brain slices. (**B**,**C**) Graphs display the median with the interquartile range. ns: not significant, * *p* < 0.05, ** *p* < 0.01, *** *p* < 0.001, and **** *p* < 0.0001 indicate significant differences with the Kruskal–Wallis test adjusted for multiple comparisons. (**D**) Normal QQ plots of the area, perimeter, circularity, and solidity for the male and female mGluR5^+/−^ animals showing the distribution deviation from normality, represented by the red line through the origin. The slice counts for each group are as follows: P14 mGlur5^+/+^ 3 slices, P28 mGlur5^+/+^ 2 slices, P14 mGlur5^+/^^−^ 4 slices, 2 male and 2 female, and P28 mGlur5^+/^^−^ 3 slices, 2 male and 1 female.

For a more in-depth analysis of microglia, we analyzed the cells on an individual and population level. We quantified the area, perimeter, circularity, and solidity of each microglial cell. We then compared the mGluR5^+/+^ and mGluR5^+/^^−^ groups at P14 and P28 and the mGluR5^+/^^−^ groups at P14 and P28 for both sexes (Figure 4). The normal quantile–quantile (QQ) plots for each individual feature when comparing the mGluR5^+/+^ and mGluR5^+/^^−^ groups show that all features are non-normally distributed; the area, perimeter, and circularity measurements have a larger deviation from a normal distribution than the solidity measurements (Figure 4A). Additionally, we see a separation in data based on age more so than genotype. For example, in area and perimeter, the P28 groups are closer to the line of a normal distribution, while both the mGluR5^+/+^ and mGluR5^+/^^−^ groups are further from the line of normality. However, when comparing circularity, the P14 groups are closer to a normal distribution than the P28 groups. Solidity cannot be differentiated based on age.

When the geometric features of each cell were analyzed and compared across the mGluR5^+/+^ and mGluR5^+/^^−^ groups, differences were observed between ages across all genotypes (Figure 4B). The P28 pups have a statistically increased area and perimeter and a statistically decreased circularity and solidity for both the mGluR5^+/+^ and the mGluR5^+/-^ groups. These data correlate with increased branching complexity occurring during aging. Meanwhile, at P14 between mGluR5^+/+^ and mGluR5^+/^^−^, there was no significant difference in the area, perimeter, circularity, and solidity. However, at P28, there was a significant increase in the area (*p* < 0.0001) and perimeter (*p* < 0.0001) and a significant decrease in both circularity (*p* < 0.0001) and solidity (*p* < 0.01). These data suggest that although the branching complexity is mainly occurring due to development from P14 to P28, the mGluR5^+/-^ genotype exhibits a change in microglial features to a lesser extent.

The morphological features of the P14 and P28 mGluR5^+/-^ animals also show sex-based differences in microglial reactivity (Figure 4C). Between the male and female pups, we see reverse trends at P14 and P28. For the area, the P14 female has a significantly greater value (*p* < 0.0001) than the P14 male, but at P28, the female cells have significantly decreased areas (*p* < 0.0001). With perimeter measurements at P14, the female pup has a significantly larger perimeter (*p* < 0.001) on average than the male pup, but at P28, the trend is reversed. In both the circularity and solidity, at P14, there is no significant difference between the male and female pups. At P28, the female pup is significantly more circular (*p* < 0.0001) and has a higher solidity (*p* < 0.0001). When comparing the change across sex during aging, microglia in the male brain experience greater extents of change between P14 and P28 compared to the female brain. Our data show a *p*-value < 0.0001 across the area, perimeter, circularity, and solidity for the male brain, no significant difference in the area and solidity for the female brain, and only a small significant difference between sex in the perimeter (*p* < 0.05) and in the circularity (*p* < 0.01).

When analyzing only the mGluR5^+/^^−^ groups, sex-based trends are visible in the normal QQ plots (Figure 4D). For each normal QQ plot, the male P14 and P28 groups have the largest separation. The male P28 pups are the closest to a normal distribution of all groups, and the P14 pups are the furthest from a normal distribution of all groups for both the area and perimeter. Similar to comparing the mGluR5^+/+^ and mGluR5^+/-^ genotypes, this trend is reversed in the circularity and indistinguishable in the solidity measurements.

**Figure 5:**
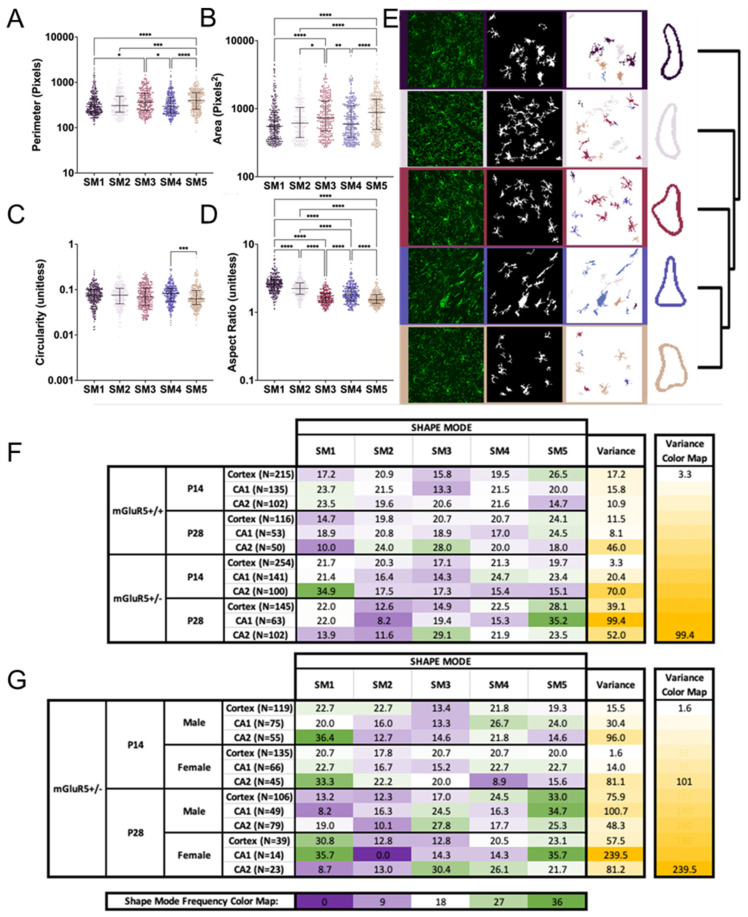
Cell shape mode (SM) parameters compared to geometric features for all groups. (**A**–**D**) Morphology parameters for the five SMs: (**A**) perimeter, (**B**) area, (**C**) circularity, and (**D**) aspect ratio. The graphs depict the median with the interquartile range. ns: not significant, * *p* < 0.05, ** *p* < 0.01, *** *p* < 0.001, and **** *p* < 0.0001 indicate significant difference with the Kruskal–Wallis test adjusted for multiple comparisons. (**E**) Representative images of the segmentation procedure shown with the original cell image, mean threshold images of segmented cells, and color-labeled cells by shape mode for visual representation as represented by the dendrogram on the right. (**F**) Global heat map of percent SM frequency for both the mGluR5^+/+^ and mGluR5^+/-^ groups at both P14 and P28 with the calculated variance of each row on the right. The 3-color heat map, green-white-purple, represents 0–18–36%, as shown by the shape mode frequency color map. The 2-color variance heat map, white-yellow, represents 3.3–99.4 variance. The N number represents the total number of cells analyzed in that group. (**G**) Global heat map of percent SM frequency for the P14 and P28 mGluR5^+/-^ groups in both the males and females. The 3-color heat map, green-white-purple, represents 0–18–36%, as shown by the shape mode frequency color map. The 2-color variance heat map, white-yellow, represents 1.6–239.5 variance. The slice counts for each group are as follows: P14 mGlur5^+/+^ 3 slices, P28 mGlur5^+/+^ 2 slices, P14 mGlur5^+/-^ 4 slices, 2 male and 2 female, and P28 mGlur5^+/-^ 3 slices, 2 male and 1 female.

To further supplement the individual microglial analysis, we used principal components analysis and k-means clustering to train a model that describes the five most-common shapes of all the data and then tested that for each age and genotype (Figure 5). For increased explainability, each of the five shape modes (SMs) are plotted against four geometric features: perimeter, area, circularity, and aspect ratio (Figure 5A–D). Representative images of the segmentation process included the original cell image, the segmented image with the mean threshold, and the color-coded image (Figure 5E). The representative images were selected because they had a high prevalence of the associated SM. The SM vs. geometric feature plots show us that the circularity of the cells played a minimal role in how the principal components analysis and k-means clustering split groups except between SM4 and SM5. The aspect ratio played a large role in differentiating all groups except SM3 vs. SM5.

When looking across features and comparing to the dendrogram and representative images, SM3 and SM5 have aspect ratios closest to 1—or a square ratio. However, SM3 and SM5 are non-significantly differentiated by the geometric features plotted; this indicates to us that the principal components analysis and k-means clustering is capturing some other aspect of the cells. From the dendrograms and representative images, both shape modes appear to have a circular shape with radial branching. SM1 has the largest aspect ratio followed by SM2, suggesting both cells are elongated. SM1 and SM2 are non-significantly different in any other shape feature, but SM2 trends towards a larger area. Finally, SM4 is ranked in the middle with regard to the aspect ratio and has one of the lowest areas and perimeters. SM4′s shape feature results compared to the representative dendrogram and representative images show elongated cells with shorter branching.

After analyzing what the SMs mean with representative images and basic geometric features, the shape mode frequencies for each group were visualized with a heat map and the variance calculated (Figure 5F,G). When comparing mGluR5^+/+^ and mGluR5^+/-^ at both P14 and P28, the variance is the highest in the P28 mGluR5^+/-^ animals. Additionally, the P14 mGluR5^+/-^ cortex shows the lowest variance and, subsequently, the most even distribution of all five SMs. By looking at individual shape modes in the P14 and P28 mGluR5^+/+^ animals, an SM shift from SM1 to SM3 and SM5 can be seen across regions during development from P14 to P28. For the comparison within the P14 and P28 mGluR5^+/-^ animals and across sexes, the variance increases with age for both animals. However, the female P14 group had the lowest variance overall, and the female P28 CA1 brain region had the highest variance. This high variance could be due to the low cell count included in the analysis. When looking at the SM frequencies, we see an inverse trend in SM1 and SM3 between the male and female microglia at P28, where the female microglia express higher frequencies of SM1 in the cortex and CA1 regions and the male microglia express higher frequencies of SM3 in the CA1.

### 3.4. Extracellular Matrix Protein Expression in mGluR5^+/-^ Pups

Based on the profiles of inflammation observed through mRNA expression levels and immunofluorescent imaging and to better understand how mGluR5 dysregulation disrupts the extracellular environment, we characterized the association between mGluR5 disruption and extracellular matrix (ECM) protein expression. We quantified the mRNA expression of five key ECM proteins for both the P14 and P28 age groups. Specifically, we looked at aggrecan (ACAN), matrixmetalloprotease-9 (MMP9), tenascin (TNR), neurocan (NCAN), and Hyaluronan and Proteoglycan Link Protein 1 (HAPLN1).

**Figure 6:**
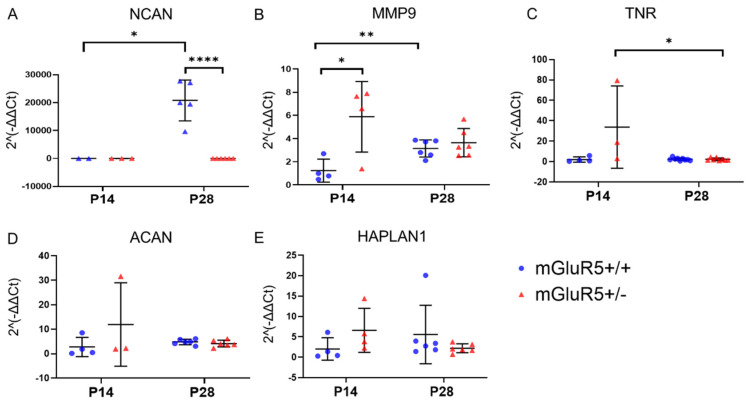
Gene expression for both P14 and P28 mGluR5^+/+^ and mGluR5^+/-^ pups are depicted for: (**A**) NCAN (n = 3 P14 mGluR5^+/+^, n = 3 P14 mGLuR5^+/-^, n = 5 P28 mGluR5^+/+^, n = 6 P28 mGluR5^+/-^); (**B**) MMP9 (n = 4 P14 mGluR5^+/+^, n = 4 P14 mGLuR5^+/-^, n = 6 P28 mGluR5^+/+^, n = 6 P28 mGluR5^+/-^); (**C**) TNR (n = 4 P14 mGluR5^+/+^, n = 3 P14 mGLuR5^+/-^, n = 6 P28 mGluR5^+/+^, n = 6 P28 mGluR5^+/-^); (**D**) ACAN (n=4 P14 mGluR5^+/+^, n = 3 P14 mGLuR5^+/-^, n = 6 P28 mGluR5^+/+^, n = 6 P28 mGluR5^+/-^); and (**E**) HAPLN1 (n=4 P14 mGluR5^+/+^, n = 4 P14 mGLuR5^+/-^, n = 6 P28 mGluR5^+/+^, n = 6 P28 mGluR5^+/-^). All gene expression results are normalized to mGluR5^+/+^ pups. Blue represents mGluR5^+/+^, and red represents mGluR5^+/^^−^. * *p* < 0.05, ** *p* < 0.01, **** *p* < 0.0001.

We observed an increase in the expression of NCAN, with a 1000+-fold increase in expression when comparing the P14 and P28 age groups of the mGluR5^+/+^ animals (*p*-value = 0.0127). A 1000+-fold decrease was observed in the P28 animals when comparing the mGluR5^+/+^ and mGluR5^+/-^ groups with a *p*-value < 0.0001 (Figure 6A). When looking at MMP9 expression, we observed a 2.5-fold increase in mGluR5^+/+^ animals from P14 to P28 of MMP9 expression with a *p*-value of 0.0082, while no significant differences across age groups were observed in the mGluR5^+/-^ pups. A 4.75-fold increase in expression was observed in mGluR5^+/-^ animals when compared to the mGluR5^+/+^ animals with a *p*-value of 0.0267 (Figure 6B). Another trend observed was a 15-fold decrease of the gene expression of TNR in the P28 age group when compared to the P14 age group in the mGluR5^+/-^ pups (*p* = 0.0317), and this was not observed in the mGluR5^+/+^ animals (Figure 6C). No statistically significant differences were observed in ACAN and HAPLN1 expression (Figure 6D,E).

### 3.5. Perineuronal Net Formation

**Figure 7:**
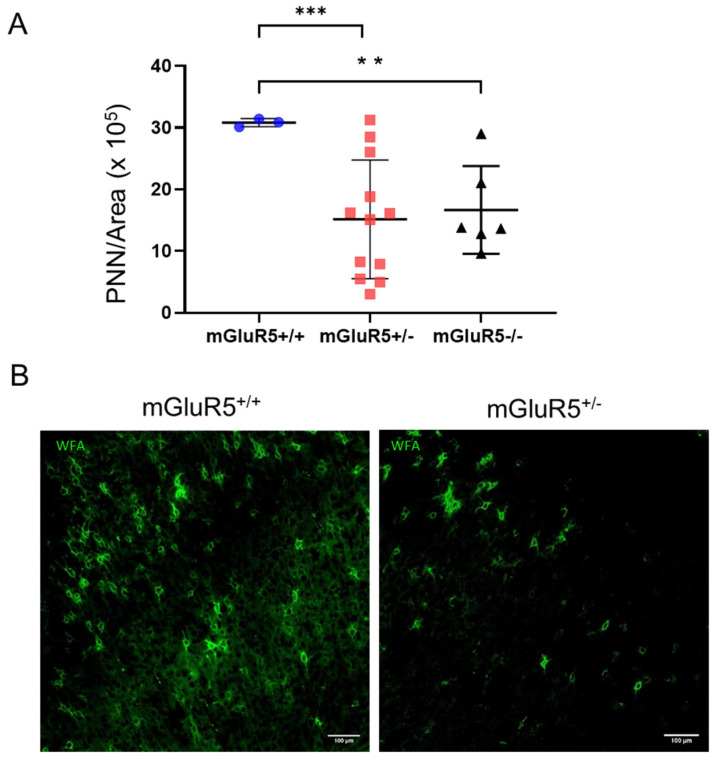
(**A**) PNN density in the cortex of P28 female mGluR5^-/-^ (n = 6), mGLuR5^+/-^ (n = 12), and mGluR5^+/+^ (n = 3) pups. (**B**) The 1000 × 1000 µm representative images of WFA+ PNNs in mGluR5^+/+^ and mGluR5^+/-^ female pups at P28. Scale bar: 100 µm. ** *p* < 0.01, *** *p* < 0.001.

PNN density was quantified using immunofluorescent methods in the P28 age group, when significant PNNs are expected to have formed [26], for mGluR5^+/+^, mGluR5^+/-^, and mGluR5^-/-^ sex-matched pups (Figure 7A). A two-fold decrease in PNN density was observed for the mGluR5^+/-^ animals when compared to the mGluR5^+/+^ animals (*p* = 0.0001). A 1.8-fold decrease was observed when comparing mGluR5^-/-^ PNN density with mGluR5^+/+^ (*p* = 0.0044).

### 3.6. Behavioral Testing

**Figure 8:**
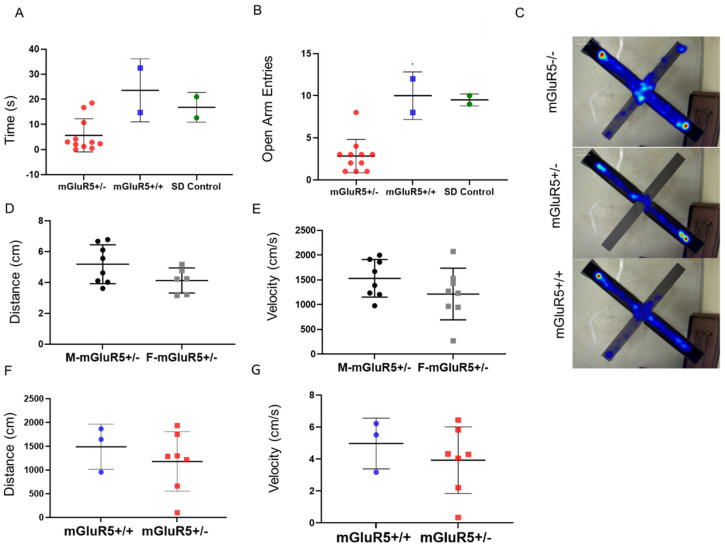
(**A**) Total time (s) spent in open arms of the elevated plus maze (n = 11 mGluR5^+/-^, n = 2 mGluR5^+/+^, n = 2 SD Control). (**B**) Total number of entries into the open arm (n = 11 mGluR5^+/-^, n = 2 mGluR5^+/+^, n = 2 SD Control). (**C**) Overlaid heat maps of time spent in various zones for mGluR5^+/+^, mGluR5^+/-^, and mGluR5^-/-^ pups (n = 11 mGluR5^+/-^, n = 2 mGluR5^+/+^, n = 2 mGluR5^-/-^). (**D**) Total distance moved in the elevated plus maze of male and female mGluR5+/- pups (n = 8 M, n = 8 F). (**E**) Average velocity (cm/s) of the pup center point in the elevated plus maze of male and female mGluR5+/- pups (n = 8 M, n = 6 F). (**F**) Total distance (cm) moved in the open field test (n = 3 mGluR5^+/+^, n = 7 mGluR5^+/-^). (**G**) Average center point velocity (cm/s) in the open field test (n = 3 mGluR5^+/+^, n = 7 mGluR5^+/-^).

Open field and elevated plus maze tests were used for neuropsychiatric hallmarks of FXS, including hyperactivity and anxiety. mGluR5^+/-^ and mGluR5^+/-^ pups in the P28 age group only were tested. SD pups were included as an external control, and no significant difference was observed between mGluR5^+/+^ and SD pups. There was a decrease in total time spent in open arms when comparing the mGluR5^+/-^ groups with both the SD animals and the mGluR5^+/+^ animals (Figure 8A). There was also a decrease in the total number of entries into the open arms for the mGluR5^+/-^ animals compared to the mGluR5^+/+^ and SD control (Figure 8B). Figure 8C shows overlaid heat maps of the paths taken by mGluR5 animals of all three genotypes in the elevated plus maze. The total time spent in the exposed arms of the elevated plus maze and number of entries into the open arm are accepted behavioral indicators of increased anxiety, as we observed in the mGluR5^+/-^ pups. Additionally, when comparing male and female mGluR5^+/-^ pups in the elevated plus maze, on average, the male mGluR5^+/-^ animals had a higher center point velocity and higher average distance moved. There was no observable difference between the mGluR5^+/+^ and the non-genetically modified SD pups (Figure 8A,B). While no quantitative conclusions can be drawn from behavioral trials including mGluR5^-/-^ animals due to the low number of animals available at these ages, extreme hyperactivity was seen when performing behavioral testing in these animals (Figure 8C). No statistically significant differences were seen when comparing total distance moved in the elevated plus maze of male and female mGluR5^+/-^ pups or average velocity of the pup center point in elevated plus maze of male and female mGluR5^+/-^ pups (Figure 8D,E), although both of these groups trended towards increased activity in male pups. No significant differences were observed in the open field test (Figure 8F,G), an assay used to assess anxiety levels and willingness to explore [27].

## 4. Discussion

Brain size and brain-to-body weight abnormalities are reported in close to 20% of autistic children [28], and abnormal birth weights and rates of growth are indicated in males diagnosed with FXS [29]. Characterization of the mGluR5^-/-^ mouse model has shown the weights of adult male mice to be significantly reduced by the absence of mGluR5, while ad libitum food intake remained constant [30]. These findings were replicated in our study in the downward trend of normalized body weight in mGluR5^+/-^, mGluR5^+/+^, and mGluR5^-/-^ pups in both the P28 and P14 age groups. The comparison of the deviation from the average litter weight by sex is shown in Supplemental Figure S2A,B, although it is noteworthy that very few mGluR5^-/-^ animals were able to survive to the P28 timepoint due to phenotypic severity. The animals that did reach this timepoint were exclusively female, leading to speculation that the mGluR5^-/-^ phenotype is so severe in male rats that they do not often survive to adulthood.

A downward trend in brain-to-body-weight ratios was also observed in our P14 pups, similar to the trends of decreasing brain volume that have been observed in neuroligin3 mGluR5^-/-^ mice, a model of another monogenic heritable form of autism [31]. Another longitudinal study performed on a model of Rett syndrome, a neurodevelopmental X-linked disorder, showed smaller total brain volumes for multiple age groups [32]. Saywell et al. specifically showed a decrease in ventricular volume as being negatively correlated with improved locomotive activity. Thus, the decreasing brain weight trends seen in our study in the mGluR5 pups aligns with those observed in other neurodevelopmental X-linked disorders.

The cytokine profiles of individuals with FXS have been demonstrated to be significantly different from typically developing controls in previous studies [33], as similarly demonstrated in this model. CXCL1 and CXCL2 are mast cell and macrophage chemokines that control the early stages of neutrophil influx during an inflammatory immune response [34]. Previous studies have shown no increase in CXCL1 and CXCL2 in adult mGluR5 knockout mice [35]; therefore, we sought to evaluate if expression differs in early brain development by characterizing these chemokines in younger age groups. The chemokines CXCL1 and CXCL2 are involved in inflammasome activation, so the increased expression in the P28 age group of mGluR5^+/-^ could demonstrate an impaired immune response in early stages of development in FXS. This dysregulation could cause increased sensitivity to neurologic damage from infection or other sources, particularly during early development.

Another key finding from this part of the study was the upregulation observed in GCPII. GCPII has been implicated in modulating extracellular concentrations of glutamate, and GCPII activity is associated with cognitive function and neuronal health [36]. GCPII is known for its ability to catalyze the hydrolysis of glutamate and N-acetyl aspartate (NAA) from N-acetyl-I-aspartyl-I-glutamate (NAAG) [37] and is also an endogenous agonist for mGluRs. The disruption of glutamate homeostasis displayed by GCPII overexpression could be an indicator of neuroinflammation in this age group. Upregulation of GCPII has also been observed in studies on experimental autoimmune encephalomyelitis (EAE), the most-used experimental model for human inflammatory demyelinating disease [38]. This is relevant in the context of the mGluR5 model because it supports the involvement GCPII upregulation in clinical impairment and axonal damage in inflammatory diseases of the CNS and demonstrates that the neuroinflammatory pathway we are investigating here is present in other disease models.

The pro-inflammatory cytokines IL-1, IL-6, IL-10, and TNFα have been shown to modulate responses in the CNS and alter neurodevelopment, impacting behavior [39]. These cytokines are regulated by numerous mechanisms [40], and the specific impact of mGluR5 antagonism on these cytokines is unknown. No significant differences between genotypes were observed for these cytokines, although the shift in cytokine expression profiles observed between the P14 and P28 age groups could support the hypothesis that the brain has different thresholds of sensitivity to neurologic damage at earlier stages of development. A detailed cytokine-mediated mechanism has yet to be conclusively determined in FXS, but the evidence of abnormal immunological functioning demonstrated in this study suggests that neuroimmune interactions may be altered in individuals with FXS at different stages of brain development.

The disruption observed in GCPII expression prompted further investigation into how excessive activation of the glutamate system could impact other hallmarks of neuroinflammation, including microglial cells, which make up the main form of active immune defense in the central nervous system. Recent studies have implicated that GCPII upregulation and impaired glutamate homeostasis are correlated with activated microglia [41]. The activation of mGluR5 has been shown to attenuate microglial activation [42]. Microglial cells are associated with many normal physiological processes, including neural development, synaptic plasticity, and cognition. Previous studies have provided evidence linking the expression of mGluR5 to pathological hallmarks of ASDs and FXS, indicating that signaling components have a role in the regulation of microglial number and activation during development [43]. Hindrance of the microglia’s ability to perform their physiological and defensive duties is fundamental in the development of ASDs [8].

Our analysis of microglial morphological features showed sex-, genotype-, and developmental-age-dependent changes. In assessing individual microglia’s morphology, the P28 mGluR5^+/-^ pups exhibit the highest area and perimeter with the lowest circularity and solidity. The geometric changes in the mGluR5^+/-^ pups could be correlated with a higher reactive state where branching increases and swelling occurs. The male pups showed the highest microglial reactivity across all shape features. Males with FXS exhibit more cognitive and behavioral problems than females, and the majority of diagnosed males meet the criteria for severe intellectual disability [44]. In a population analysis of microglial shapes, the P14 mGluR5^+/-^ male exhibits higher variance than the female in SM frequency, with a lower expression of SM3 and slightly higher expression of SM4. The differences from the female SM frequencies suggest a sex-based preference for certain microglial phenotypes.

The population trends analyzed with VAMPIRE also support the conclusion that the male mGluR5^+/-^ pups exhibited a more severe phenotype that is exacerbated at P28. The P28 male mGluR5^+/-^ pups exhibited higher frequencies of SM5 and SM3—the shapes with the aspect ratio closest to 1 and the largest areas. In comparison, the P28 female mGluR5^+/-^ pups exhibited the highest frequencies in SM5 and SM1, an SM correlated with the highest aspect ratio. It is possible that the SM5 and SM3 combination in the male pups causes more severe outcomes than the SM5 and SM1 combination in the female. We also observed an increase in the microglia density in the cerebral cortex of male mGluR5^+/-^ P14 pups. The increase in microglia density observed in male rats in this model could be correlated with an ongoing neuroinflammatory process contributing to the more severe phenotype observed in male subjects with FXS.

Globally, we observed an increase in microglia density in the P14 pups when comparing the two different mGluR5^+/-^ age groups, which may correlate with an increased inflammatory state in early brain development, consistent with the patterns we observed in the cytokine expression profiles. We also observed a decrease in the total area covered per individual microglial cell in the P14 age group for both genotypes. Activated microglia take on an amoeboid state as opposed to a surveying ramified state [45]; thus, a greater area covered per cell could indicate a higher level of activation in early brain development. The increase in microglia density found in the mGluR5^+/-^ pups in our model is indicative of an inflammatory response, implicating the neuroprotective properties of mGluR5.

Disruption of the normal microglial phenotype has been associated with dysregulation of the ECM. Both ACAN and HAPLN1 have been demonstrated to play an important role in the organization of the neuronal extracellular space via binding hyaluronic acid and have been implicated in regulation of neural plasticity [46]. MMP9 has been shown to degrade ECM proteins and regulate tissue remodeling and has been shown to be upregulated in models of FXS. TNR is an ECM glycoprotein expressed during embryonic CNS development. It is present at lower levels in adults, but reappears under pathological conditions and in neurodegenerative disorders [47,48]. Thus, we wanted to see if expression was increased in either age group of the FXS model. NCAN is a component of the ECM matrix in the brain and has been shown to inhibit neuronal adhesion and play a role in axon guidance and neuron growth [49]. Previous studies have shown that elimination of NCAN led to reduced inhibitory synapses [50]; therefore, it is of interest to analyze the impact of the elimination of NCAN on the formation of ECM in the mGlurR5 model. TNR levels have been shown to be upregulated during times of stress and in pathological states, so the upregulation in P14 mGluR5^+/-^ could indicate that this age group is under a greater degree of neuroinflammatory stress than the P28 age group or P15 mGluR5^+/+^.

MMP9 is an important molecule in central nervous system development and plasticity and has been implicated in several neurodegenerative disorders [51]. MMP9 has been found in many cell types including glial cells [52], and MMP9 translation has been shown to be suppressed by binding to FMRP, implicating a role of MMP9 in FXS [53]. mGluR activation causes the dissociation of FMRP from MMP9 mRNA and the subsequent synthesis of the MMP9 protein [53]. It has been shown in mouse models for FXS that levels of MMP9 were increased and this increase was negatively correlated with behavioral improvement in young mice in general cognition and anxiety [54]. Thus, the overexpression of MMP9 that is observed across the P14 age group could be correlated with a decrease in cognitive performance.

A maladaptive ECM structure has been associated with neurodegenerative conditions, as the ECM plays a vital role in neuronal plasticity [55]. Thus, downregulated expression of ECM proteins in the P14 age group could implicate abnormal synaptic plasticity that underlies neuropsychiatric disorders. To investigate this further, we quantified perineuronal net (PNN) expression in the P14 and P28 animals. PNNs are specialized ECM structures that are responsible for stabilizing synapses in developed brains [56]. The decrease in PNN density observed in both the mGluR5^+/-^ and mGluR5^-/-^ animals indicates changes in synaptic plasticity and neuronal function when compared to the wild-type animals, as well as reduced protective barriers against oxidative stress and neurotoxins.

Due to changes observed in brain cell morphology, cytokine expression, and ECM, we investigated if these changes at the molecular and cellular level would translate into measurable behavioral differences like those that would be used for a clinical diagnosis of FXS. In children who are diagnosed with FXS, especially males, a commonly noted behavioral feature is attentional issues and anxiety [57]. Decreased total time spent in the exposed arms of the elevated plus maze and number of entries into the open arms are accepted behavioral indicators of increased anxiety, as we observed in the mGluR5^+/-^ pups (Figure 8A,B). No significant differences were observed in the open field test (Figure 8F,G); an assay used to assess anxiety levels and willingness to explore [27]. However, our results trended towards decreased exploration and movement in the mGluR5^+/-^ animals. Our studies involved too few animal numbers due to colony closure, an unfortunate outcome of lab shutdown for unknown time periods at the onset of the COVID-19 pandemic. A next step for FXS models could be to further investigate these behaviors with other measures of anxiety with enough animal numbers to appropriately power the study to identify significant differences based on age, sex, and genotype.

## 5. Conclusions

MGluR5 inhibition negatively impacts growth and development patterns, stimulates microglial activation in vivo, disrupts cytokine expression, creates a maladaptive ECM structure, and influences observable behavior. The changes in cytokine expression observed in this model along with the reduced density of PNN formation indicate that mGluR5 suppresses inflammatory signaling and can downregulate microglial activation and neuroinflammation. Additionally, the increased heterogeneity of gene expression in the heterozygous knockout animals reflects complicated interactions between neurodevelopmental and immune processes in ASDs, as well as variance in global levels of gene expression. Gene-specific studies are beneficial for analyzing the underlying basis of a disease such as FXS that interacts with multiple systems, but global levels of gene expression regulation can result in phenotypic variance seen both in the clinical model of FXS [58] and in this model with hallmarks of FXS.

Heterozygous deletion of the mGluR5 receptor profoundly impacted multiple markers of neuroinflammation in this model, including pro- and anti-inflammatory cytokine expression, GCPII expression, microglia density and morphology, behavioral data, and ECM protein markers. The mGluR5^-/-^ animals had significantly worse phenotypic outcomes, and few survived to ages beyond P14, bolstering the hypothesis that the heterozygous deletion has greater clinical relevance. The present results characterizing microglia across age groups, sex, and genotypes highlight the role that the mGlur5 receptor plays in determining the morphological properties of microglia. Additionally, the increase in variance observed in the cytokine datasets and population trends in microglia SMs could potentially represent phenotypic variation commonly observed in models of ASDs and in clinical cases of ASDs. Statistical analysis of the GCPII and CXCL datasets indicated increased variance of phenotypic severity in the mGluR5^+/-^ animals. Recent clinical trials of FXS have revealed concerns around phenotypic variance in the lack of biomarkers that can track whether a specific mechanism is responsive to a new drug and whether the response correlates with clinical improvement [59]. The results generated in this study can contribute to establishing a phenotypical baseline and identifying biomarkers over multiple domains that can be identified at specific stages of development. The detectable alternation of neurotransmitter systems displayed in these data highlights the importance of developing animal models that can help define diseases that have poorly understood and complex mechanisms of disease development and progression.

## Data Availability

The data presented in this study are available upon request from the corresponding author.

## Supplementary Materials

The following Supporting Information can be downloaded at: https://www.mdpi.com/article/10.3390/life12091308/s1, Figure S1: Total body weight of mGluR5^+/+^ animals; Figure S2: Sex comparisons for deviation from litter-matched body weight.
