# Supplementary material for: Characterization of a mGluR5 Knockout Rat Model with Hallmarks of Fragile X Syndrome
Source: Life (Basel). 2022 Aug 25;12(9):1308. doi: 10.3390/life12091308 (PMC9504063; doi:10.3390/life12091308)
Supplement: Supplementary file 1 [file life-12-01308-s001.zip › life-1818395-supplementary.pdf]

Supplemental Figures

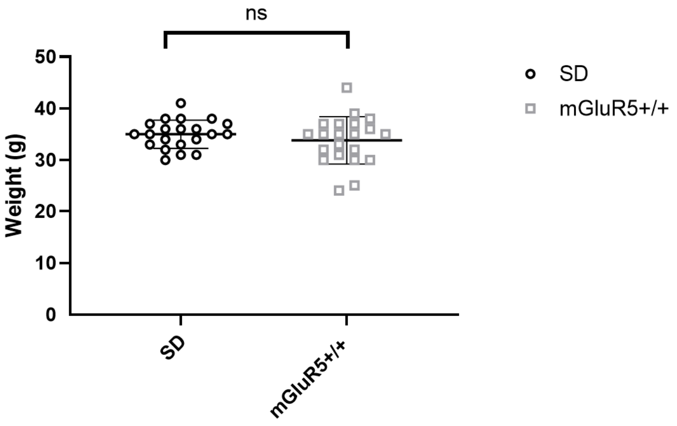

Figure S1. Total body weight in grams of mGluR5$^{+/+}$ (n=20) animals compared to SD control (n=21) animals for the P14 age group.

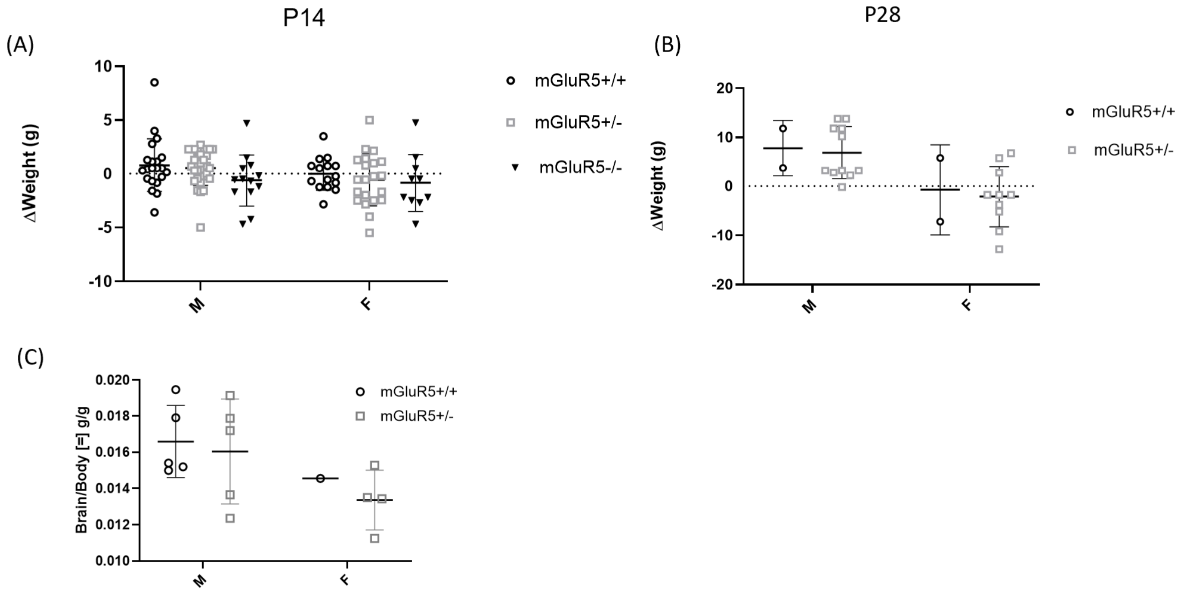

Figure S2. (A) Deviation from average litter matched body weight with sex comparison for P14 male mGluR5$^{+/+}$ (n=22), mGluR5$^{+/-}$ (n=37), mGluR5$^{-/-}$ (n=13), and female mGluR5$^{+/+}$ (n=15), mGluR5$^{+/-}$ (n=20), and mGluR5$^{-/-}$ (n=10) pups. (B) Deviation from average litter matched with sex comparison for P28 age group, male mGLuR5$^{+/+}$ (n=2), male mGLuR5$^{+/-}$ (n=12), female mGluR5$^{+/+}$ (n=2), and female mGluR5$^{+/-}$ (n=11). (C) Brain to body weight ratio comparing P14 male mGluR5$^{+/+}$ (n=5), male mGluR5$^{+/-}$ (n=5), female mGluR5$^{+/+}$ (n=1), and female mGluR6$^{+/-}$ (n=4) pups.
